# Incarcerated Diaphragmatic Hernias After Roux-en-Y Gastric Bypass

**DOI:** 10.7759/cureus.33063

**Published:** 2022-12-28

**Authors:** Carolina E Garcia, Ruchir Puri

**Affiliations:** 1 Surgery, University of Florida College of Medicine – Jacksonville, Jacksonville, USA; 2 Gastrointestinal Surgery, University of Florida College of Medicine – Jacksonville, Jacksonville, USA

**Keywords:** paraesophageal hiatal hernia, s:hiatal hernia, diaphragmatic hernia repair, incarcerated hernia, laparoscopic roux-en-y gastric bypass

## Abstract

Laparoscopic Roux-en-Y gastric bypass (LRYGB) is one of the most common operations performed for morbid obesity. Some of the known surgical complications include anastomotic leaks and small bowel obstructions due to internal hernias. Diaphragmatic hernias are common in the general population, and repair of symptomatic hernias is generally recommended. Diaphragmatic hernia after a prior LRYGB is markedly less common. Diaphragmatic hernias can occur via a hiatal defect or rarely a parahiatal defect that is found lateral to the hiatus. We present two cases of incarcerated diaphragmatic hernias after a LRYGB with vastly different presentations. The first patient presented with a giant defect containing incarcerated jejunum after a prior LRYGB. The second patient presented with a parahiatal defect with an incarcerated remnant stomach. The first patient was successfully managed laparoscopically by reinforcing the defect with a mesh after defect closure. The second patient required an open operation due to the inability to reduce the tightly incarcerated stomach and defect approximated with sutures without the need for mesh reinforcement. Both patients did well postoperatively and remain symptom-free.

## Introduction

Laparoscopic Roux-en-Y gastric bypass (LRYGB) is one of the most common operations performed for morbid obesity and results in excellent weight loss [1]. Some of the known surgical complications include anastomotic leaks and small bowel obstructions due to internal hernias [2]. Other common complications include gastrointestinal (GI) bleeding, gastrojejunal bleeding, deep vein thrombosis, intra-abdominal abscesses, gastric remnant leaks, gastro-gastric fistulas, and incisional hernias [3]. Diaphragmatic hernia after a LRYGB, on the other hand, is rarely reported. The majority of diaphragmatic hernias in adults occur via hiatal defects, but on very rare occasions hernias occur through lateral, parahiatal defects. We present two cases of incarcerated diaphragmatic hernias after a LRYGB with vastly different presentations. Both case presentations are distinct due to the location of the diaphragmatic defect, the hernia contents, and the type of defect closure. The case presentations highlight the rarity of these cases and the urgency with which they should be repaired surgically.

This article was previously presented as a meeting poster at SAGES in 2015 and as a video presentation at the AHS meeting in 2021.

## Case presentation

Case 1

**Figure 1:**
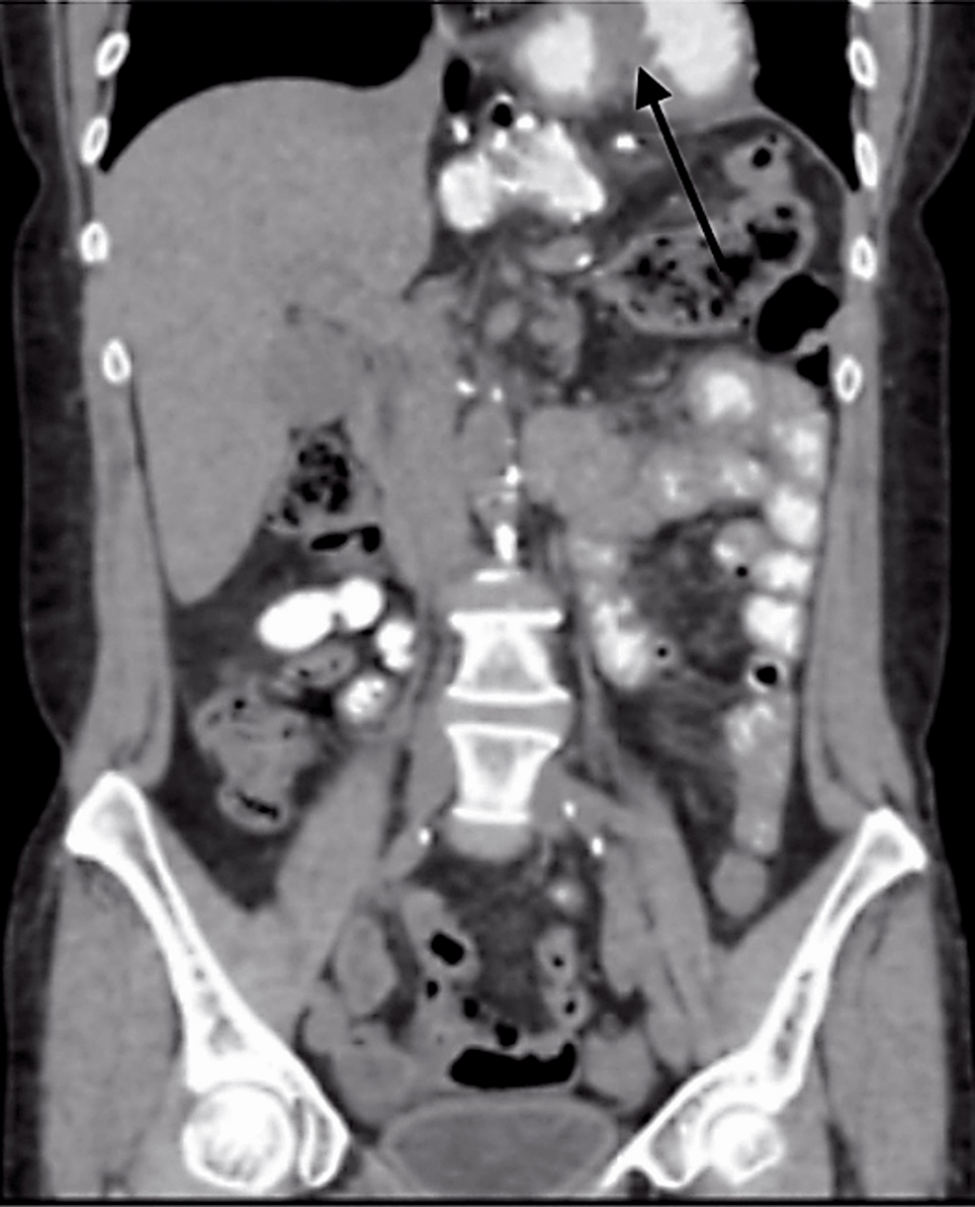
CT scan showing herniated jejunum above the diaphragm (marked with an arrow).

A 54-year-old female was referred to the clinic for evaluation of epigastric pain, nausea, and emesis for seven days. She had a significant past medical history of gastroesophageal reflux disease (GERD) and a past surgical history of a sleeve gastrectomy in 2012 that was later revised to a LRYGB for severe GERD and an abdominoplasty. She was hemodynamically stable, and on a physical examination, her abdomen was tender in the epigastric region. Her labs were unremarkable. A CT scan revealed a large paraesophageal hernia containing jejunum with concern for obstruction (Figure 1).

**Figure 2:**
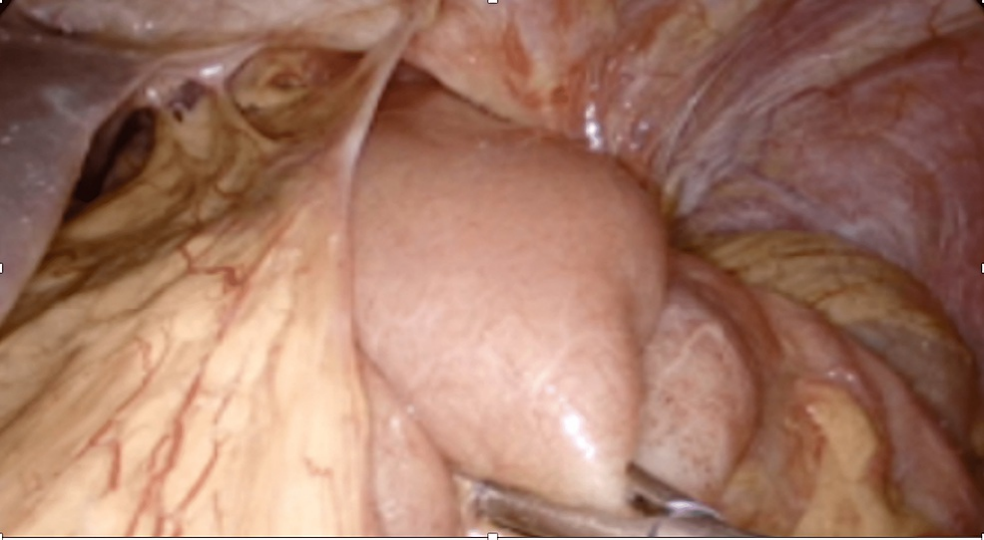
Laparoscopic view showing incarcerated jejunum through the diaphragm.

She was taken to the operating room (OR) for an emergent laparoscopic paraesophageal hernia repair. The operation was begun by reducing the jejunum into the peritoneal cavity (Figure 2).

**Figure 3:**
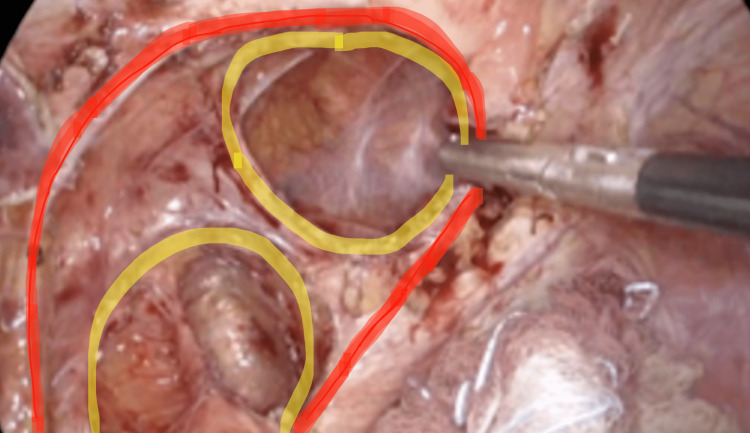
Diaphragmatic defects after reduction of the jejunum, outer defect in red and inner defects in yellow ("hole in hole" configuration).

Omental adhesions were present around the hernia defect, which were dissected using hook cautery and ultrasonic shears. Initially, it seemed that the patient had a large anterior defect within the left hemidiaphragm. The area around the defect was dissected free of all adhesions, and the diaphragmatic crura were identified. After complete reduction of the jejunum and mobilization of the omentum off the diaphragm, a giant paraesophageal hernia was revealed, with the defect measuring 7 x 6 cm. A smaller “hole in hole” type defect was noted within the mediastinum through which the jejunum was previously incarcerated (Figure 3).

**Figure 4:**
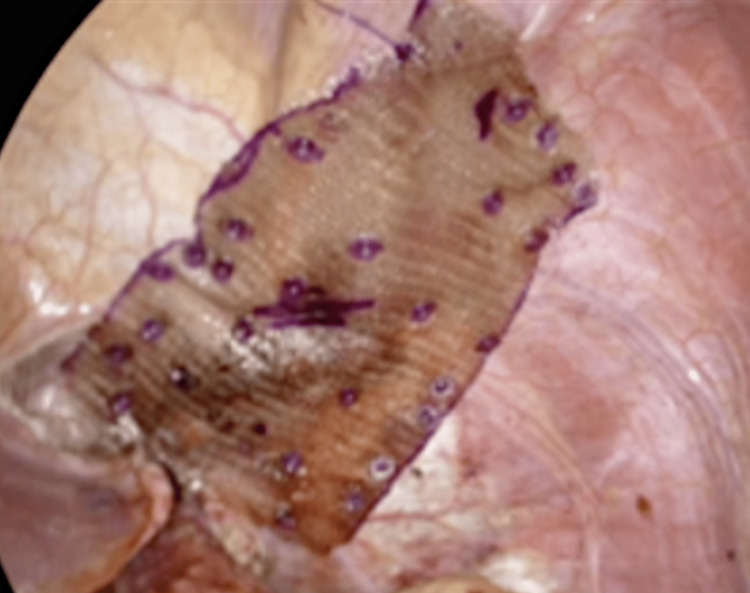
Defect after closure and mesh reinforcement.

We began the repair by dropping the intra-abdominal pressure to 8 mmHg. Multiple 0 Ethibond (Ethicon, Raritan, New Jersey, United States) sutures were used to approximate the hiatus anterior to the esophagus. Interrupted 0 silk sutures were utilized to reduce the tension off the Ethibond sutures. Given the giant defect, we elected to use a coated medium-weight polypropylene mesh to reinforce the repair. The mesh was anchored to the diaphragm using an absorbable tacking device as well as polydioxanone suture (PDS; Ethicon, Raritan, New Jersey, United States) (Figure 4). 

Her postoperative course was unremarkable. An upper GI study was performed the next day, which was negative for leaks or strictures. On postoperative day two, she was ambulating, the pain was under control, she was tolerating a clear liquid diet, she had bowel function, and she was discharged home. She was seen in the clinic three weeks after the operation, and her diet was advanced. She had an uneventful recovery. 

Case 2

**Figure 5:**
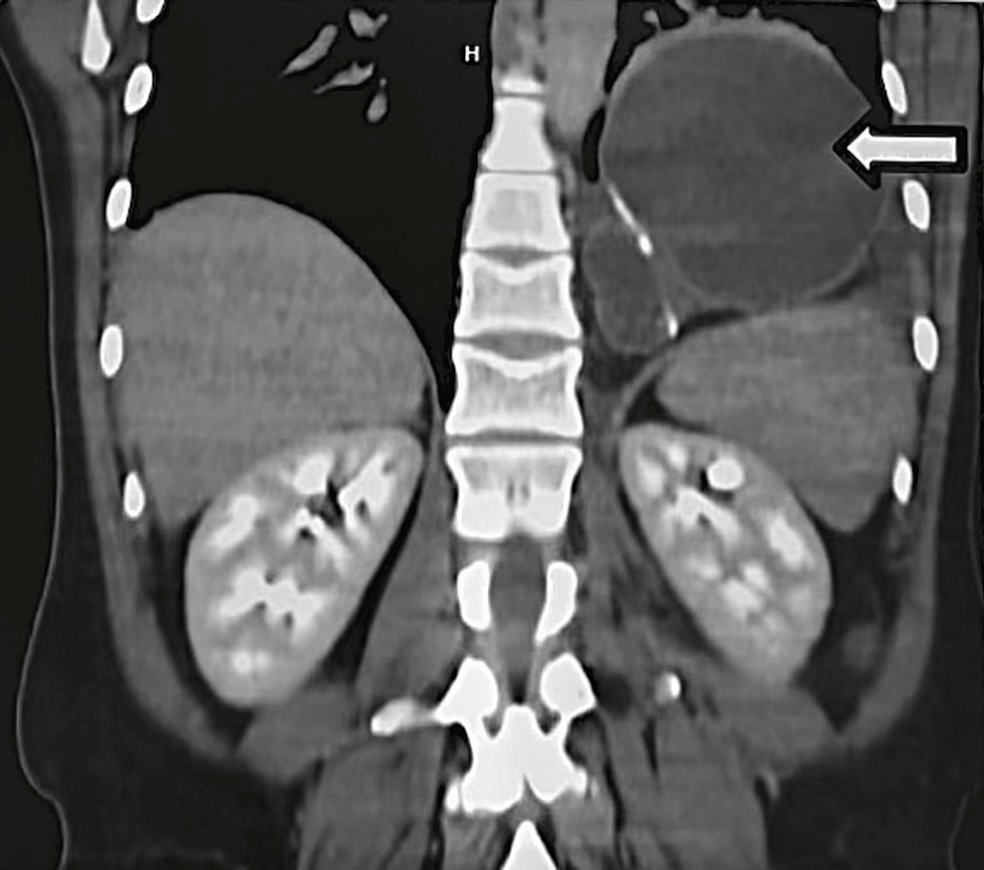
CT scan showing herniated remnant stomach in the left chest (marked with an arrow).

The second patient is a 45-year-old female who presented with two weeks of severe left chest pain that worsened over two days. The pain was associated with severe nausea and retching. She had a prior history of LRYGB done in Mexico four years ago, apparently with a hiatal hernia repair. Prior to the onset of symptoms, she was reportedly lifting a couch. She was hemodynamically stable with a benign abdominal exam. Her labs were unremarkable. A CT scan revealed the remnant stomach to be in the left chest (Figure 5). Of note, she had a CT scan two years earlier for back pain, in which no diaphragmatic hernia was noted. A preoperative swallow study confirmed the roux limb to be in an appropriate position. 

**Figure 6:**
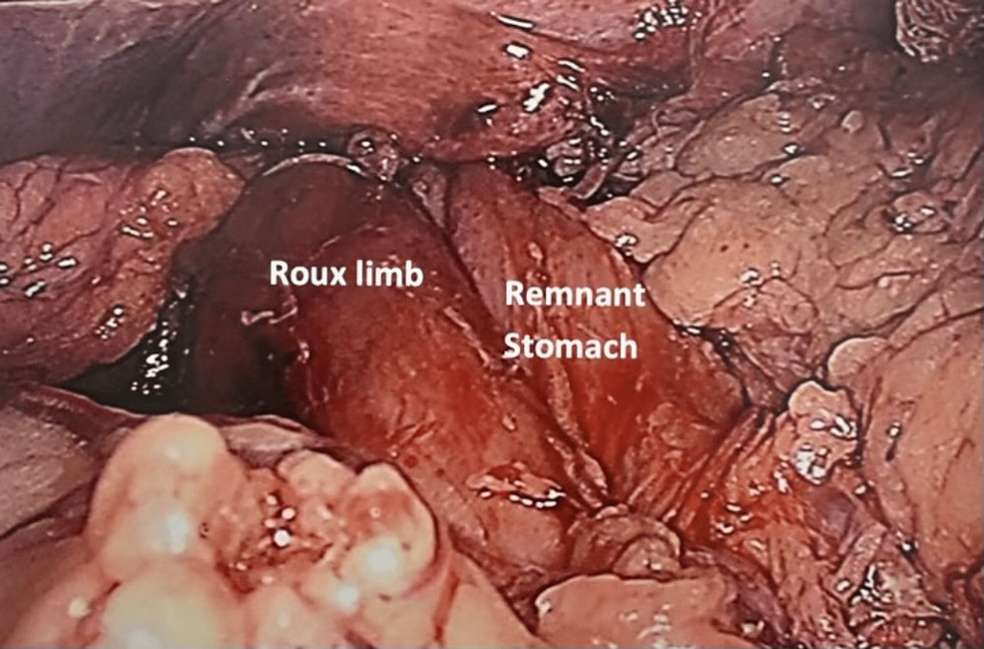
Picture showing the herniated remnant stomach to the left of the Roux limb.

She was taken to the OR for a laparoscopic repair of the left diaphragmatic hernia, where the hiatus appeared normal and the majority of the remnant stomach was incarcerated and herniated into the chest (Figure 6).

We had to convert to an open operation due to the inability to reduce the stomach laparoscopically.

**Figure 7:**
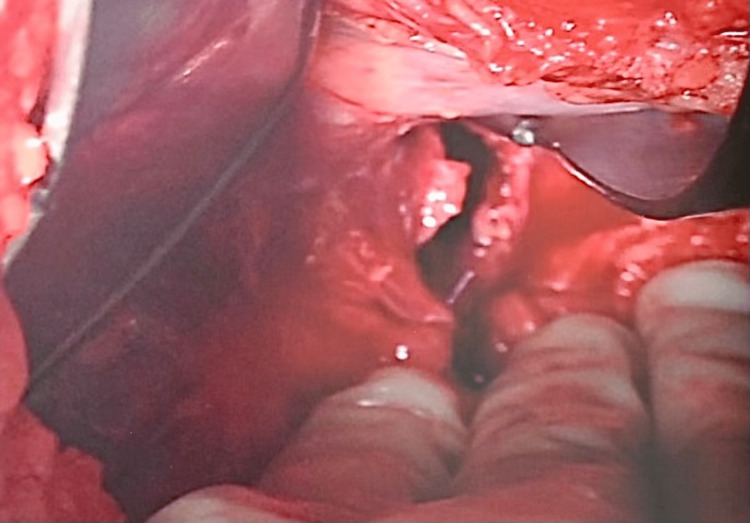
Picture showing the defect in the diaphragm after reduction of the stomach.

**Figure 8:**
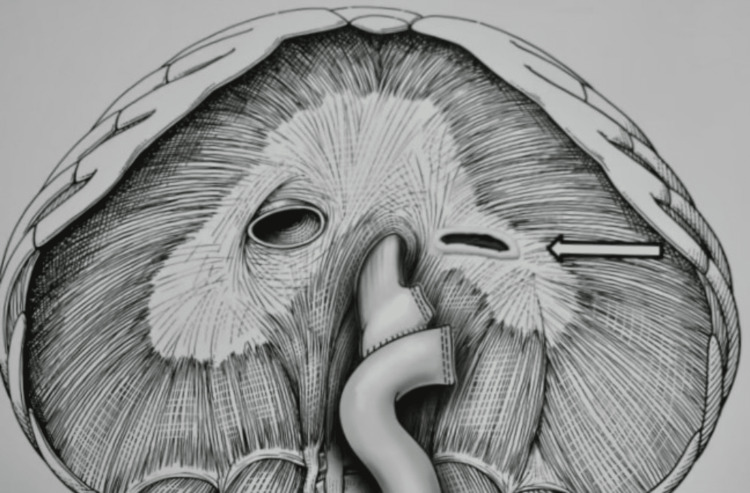
Animation showing the parahiatal hernia (marked with an arrow). Source: Author Puri R; traumatic parahiatal hernia containing remnant stomach.

The patient had a 4 x 4 cm defect, 2 cm lateral to the hiatus with minimal adhesions (Figure 7). There was no sac, suggesting recent incarceration, and the stomach was viable. The defect was closed with multiple figures of eight Ethibond sutures, and a gastrostomy tube was placed in the remnant stomach after reduction. An animation shows the anatomical location of the defect (Figure 8).

Her postoperative course was uneventful except for an asymptomatic left-sided effusion, which was managed nonoperatively. She was placed on a diet after resuming bowel function, and she was discharged home on postoperative day five. The gastrostomy tube was removed six weeks after surgery. She is a year out from her surgery and doing well. 

## Discussion

LRYGB results in excellent weight loss and ameliorates the comorbidities of obesity better than medical therapy [1]. LRYGB is a technically challenging operation, and several surgical complications have been reported. Anastomotic leaks and small bowel obstructions leading to strangulation are perhaps the most dreaded. Other common complications include GI bleeding, gastrojejunal bleeding, deep vein thrombosis, intra-abdominal abscesses, gastric remnant leaks, gastro-gastric fistulas, and incisional hernias [2,3]. Prompt surgical intervention is warranted to prevent strangulation in any incarcerated hernia. The incidence of internal hernias after LRYGB is 0.2-9% [4] and higher with the laparoscopic approach in comparison with the open approach. The etiology of increased incidence is not clear; however, fewer adhesions with laparoscopic surgery have been implicated. Despite this fact, the laparoscopic approach is favored due to decreased postoperative pain, hospital length of stay, and earlier return to activities of daily living [5]. There is a lack of robust data regarding the incidence of diaphragmatic hernias following LRYGB. Available literature suggests that diaphragmatic hernias are a rare complication after LRYGB occurring in less than 1% of cases [6]. 

These cases are unique in two respects. The first patient has a giant anterior defect, which is uncommon for typical paraesophageal hernias. Most defects are located posterior to the esophagus, and a posterior cruroplasty is performed. Since this defect was anterior, so was the diaphragmatic repair. The very large anterior defect prompted us to use a permanent mesh to minimize the risk of recurrence. For typical posterior cruroplasty following a hiatal hernia repair, a synthetic mesh is not recommended due to the risk of erosion into the esophagus [7]. As far as biologic meshes go, there has been no documented long-term benefit of using a mesh [8]. 

The second patient had a left-side diaphragmatic defect lateral to the hiatus with an incarcerated remnant stomach. Isolated left-sided defects are not commonly seen except for traumatic diaphragmatic hernias. The patients’ history suggested that this may have been preceded by trauma but what led to the diaphragmatic weakness is unclear. It is possible that she may have had a small Bochdalek hernia or a parahiatal hernia that was missed during her index operation. Bochdalek hernias are usually congenital defects in the posterolateral diaphragm, which are rarely seen in adults [9]. The stomach could not be reduced laparoscopically, and an open operation was required. Increasing the size of the defect or performing a gastrotomy to decompress the stomach could have been an option that would have allowed the entire case to be performed laparoscopically. She recovered well regardless of the approach. 

## Conclusions

Incarcerated diaphragmatic hernias are a rare complication after RYGB. These hernias can occur via hiatal or parahiatal defects and have varied presentations. These hernias are surgical emergencies and require prompt recognition and operative management to prevent intestinal ischemia. Depending on the size of the defect, hiatal hernias can be repaired with or without mesh. The laparoscopic approach is ideal for most patients and results in satisfactory outcomes.
